# Astrotourism and Night Sky Brightness Forecast: First Probabilistic Model Approach

**DOI:** 10.3390/s19132840

**Published:** 2019-06-26

**Authors:** Eleazar C-Sánchez, Agustín J. Sánchez-Medina, Jesús B. Alonso-Hernández, Augusto Voltes-Dorta

**Affiliations:** 1 Instituto Universitario de Ciencias y Tecnologías Cibernéticas (IUCTC), University of Las Palmas de Gran Canaria, Campus de Tafira, 35017 Las Palmas de Gran Canaria, Spain; 2 Instituto Universitario de Ciencias y Tecnologías Cibernéticas (IUCTC), University of Las Palmas de Gran Canaria, Despacho C-2.21, Ed. de Económicas y Empresariales, Campus de Tafira, 35017 Las Palmas de Gran Canaria, Spain; 3 Instituto para el Desarrollo Tecnológico y la Innovación en Comunicaciones (IDeTIC), University of Las Palmas de Gran Canaria, Despacho D-102, Pabellón B, Ed. de Electrónica y Comunicaciones, 35017 Las Palmas de Gran Canaria, Spain; 4 Management Science and Business Economics Group, University of Edinburgh Business School, Edinburgh EH8 9JS, UK

**Keywords:** night sky brightness, sky quality metre, astrotourism, celestial tourism, ARIMA, artificial neural network (ANN)

## Abstract

Celestial tourism, also known as astrotourism, astronomical tourism or, less frequently, star tourism, refers to people’s interest in visiting places where celestial phenomena can be clearly observed. Stars, skygazing, meteor showers or comets, among other phenomena, arouse people’s interest, however, good night sky conditions are required to observe such phenomena. From an environmental point of view, several organisations have surfaced in defence of the protection of dark night skies against light pollution, while from an economic point of view; the idea also opens new possibilities for development in associated areas. The quality of dark skies for celestial tourism can be measured by night sky brightness (NSB), which is used to quantify the visual perception of the sky, including several light sources at a specific point on earth. The aim of this research is to model the nocturnal sky brightness by training and testing a probabilistic model using real NSB data. ARIMA and artificial neural network models have been applied to open NSB data provided by the Globe at Night international programme, with the results of this first model approach being promising and opening up new possibilities for astrotourism. To the best of the authors’ knowledge, probabilistic models have not been applied to NSB forecasting.

## 1. Introduction

Tourism is one of the most expanded industries throughout the world and one of the most changing and rapidly developing economic sectors. Recently, this industry has focused on specific branches that offer multiple tourism experiences and activities [1]; among them is ecotourism, one of the fastest growing areas, which has drawn special interest. The amount of research into this discipline has increased in the last years with the appearance of new institutes, journal articles and books based on this topic. Its growth has even generated elective or core subjects as well as specific programmes in many universities [2]. This expansion has led to the creation of specialised products for different kinds of interests in what was considered a unique concept of tourism until some years ago. “According to its definition, ecotourism can involve both cultural and environmental tourism” [3] and so, celestial ecotourism can be considered in terms of “learning and the maximisation of positive ecological and sociocultural impacts” [4]. As D. Weaver notes, celestial tourism, astrotourism, astronomical tourism or, less frequently, star tourism consists in observation of celestial phenomena which can be appreciated in a natural way, excluding planetariums, which present astronomical recreations in artificial spaces, astronomy conventions or similar. A. Farajirad and P. Beiki [5] defined astronomical tourism as “when an individual interested in the sky (an amateur or professional astronomer or anyone interested in the field) travels to a location other than their residence to study and explore the wonders of the sky”. It is also considered as nature-based tourism, where visitors find it attractive travelling to natural places, mainly for two reasons: firstly, they can experience unknown environments and places, and secondly, guests find specific facilities which cannot be found in their places of origin [6]. However, only locations with the necessary conditions can be developed for this kind of tourism, therefore, destinations with dark skies at night that are free from artificial light pollution, can be a potential area for developing related tourism business. Several organisations have appeared in favour of preserving dark skies such as the “Astronomy and World Heritage” project which was created by UNESCO (United Nation Educational, Scientific and Cultural Organization) with the aim of “raising awareness of the importance of astronomical heritage worldwide and to facilitate efforts to identify, protect and preserve such heritage for the benefit of humankind” [7], the Starlight foundation by the Institute of Astrophysics of the Canary Islands [8], the Globe at Night programme [9], funded by the National Optical Astronomy Observatory of the United States, which is an international citizen-science campaign that fights for the awareness of light pollution or the International Dark-Sky Association (IDA) [10], founded in 1988, among others. These sources show multiple sources of information ranging from places where celestial phenomena can be observed to multimedia resources to interactive data maps. In 2001, IDA initiated a certification programme aimed at global communities, parks and protected areas mainly with the intention of preserving dark skies through six different kinds of designations. Likewise, the Starlight project started a certification programme for those places with excellent night sky vision. Both help to keep safe dark night places, but at the same time, they also encourage and promote astrotourism.

People’s interest in visiting specific astrotourism destinations is motivated by different celestial phenomena, which can be classified by addressing the sun’s position [4]. According to this, the classification of stars, skygazing, meteor showers or comets, among others, would be classified as nocturnal, while rainbows or solar/moon eclipses would be considered diurnal; finally, sunrises and sunsets would be treated as crepuscular phenomena. Astronomical activities can also be classified responding to market subsegments, specifically five types: visits to astronomical observatories, destinations for observing auroras, public astronomy parks with dark skies, amateur astronomy associations which offer public programmes and other providers that offer related products (e.g., private or public astronomical facilities) [11]. Understanding the visitor’s motivation is essential for focusing on the precise target group by providing solutions for their needs and therefore being able to develop and improve products and services in suitable destinations [6]. 

Simultaneously, Information and Communication technologies (ICT) and digital technologies have revolutionised the tourism industry and have introduced opportunities for developing new applications which support tourism activities. In this manner, “smart tourism aims to apply intelligent perceptions of all kinds of tourism information, such as tourism resources, tourist economics, tourism activities, and tourism participants, among others, to develop the acquisition and adjustment of real-time tourism information through mobile Internet or Internet terminal equipment” [12]. Therefore, technology should be treated as an integral part of a tourism subsector and contribute to its development by adding value to this activity. It brings the opportunity to improve tourism services (e.g., personalise features such as temperature/humidity room control according to customer preferences) or even create new services in order to offer different customer experiences (e.g., augmented reality), so that the sector becomes more professional and competitive. Moreover, technology can be considered part of everyday life and its use is increasing day by day. Proof thereof is the fact that the undertaking of some daily tasks cannot be imagined nowadays without a smartphone or a terminal with Internet access. According to this reality, private and public organisations have invested in technological platforms which support a real-time system to be accessed from any end-user device. Several investigations have been carried out in this area regarding the use of such data in an effort to model and forecast future behaviour, which is a challenging task when weather-dependent variables are predicted and which is the case of the present research. With the aim of contributing to the development of astrotourism, this research proposes an accurate method for forecasting the darkness of night skies. It should be considered that visitors usually have to travel long distances, generally in the early hours of the night, to get to suitable places for celestial tourism which are typically far away, and so, if it so happens that at the time, the sky conditions are not appropriate, they leave the place with the feeling of having “wasted their time”. Furthermore, some visitors carry special instruments for contemplating celestial phenomena and consequently this experience would have a negative impact in these cases. This research proposes a short-term forecast of night sky brightness (NSB) based on real field data with the aim of providing advance information about night sky quality in order to increase the guarantees for tourists being able to contemplate good celestial views and enjoy the experience, which is a crucial factor for encouraging client loyalty.

To perform this task, open datasets, which collect real NSB data provided by the Globe at Night programme, were used for training and testing a first model approach. The Globe at Night programme is an international programme which monitors sky brightness though a network of *Sky Quality Metres* (SQM) worldwide. This represents a first step towards the development of a tourism application that not only allows visitors to get information about celestial touristic places, but also provides them with NSB forecasts, so that it may add value and promote this growing tourist sector. Specifically, two probabilistic models, Autoregressive Integrated Moving Average (ARIMA) and Artificial Neural Network (ANN), have been trained and tested for establishing an accurate forecasting method of NSB. 

The rest of this paper is organised as follows. Section 2 performs a literature review of the previous proposed model and explains the sensor technology used for this research. Section 3 introduces the technology of the sensors used to capture the data used in this research. Section 4 briefly describes the methodological approach, while Section 5 presents the result of the research. Section 6 concludes the paper and outlines its future prospects.

## 2. Related Work and Motivation

Weather-dependent variables are challenging to forecast because of so many uncertainties that are often not expected. In this specific case, nocturnal sky brightness can be affected by clouds [13], dust particles in the atmosphere [14,15] or even wild animals in uncontrolled remote areas which can block the sensor view. Moonlight also affects the SQM measurements by reducing the NSB levels, which is not appropriate for night sky observations. Puschnig et al. [16] studied the effect of the moonlight and cloud conditions on the NSB measurements, concluding that moonlight affects the NSB values considerably, but that cloudy conditions are even brighter, especially in those places near bright cities. This means that NSB values are also affected by the lunar cycle and therefore periodicity in the NSB related to the lunar phase was observed in the measurements carried out in this paper. Additionally, the effect of the clouds on the NSB values has reversed the previous concept of darkness because of the rapid change in the use of artificial light at night in the Anthropocene epoch [17].

In this section, previous physically based models for sky brightness radiation are reviewed, as well as some probabilistic models for seasonal time series.

### 2.1. Physically Based

In 1986, Garstang [18] published a general physical model that calculates the sky brightness at a specific point on earth based on multiple light sources. This research concludes that NSB predictions are satisfactory for cities outside observers, as well as for small city centres, but the model does not perform well for big cities. Two years later, the same author published a model with the purpose of predicting NSB variation due to seasonal changes, concluding that the “results give an idea of the size of likely seasonal effects on NSB for good astronomical sites”, but more data should be used for understanding how its behaviour can be attributed to seasonal effects or short-term variations because of local causes [19]. Garstang’s light propagation model has been successfully tested [15,18,19] and used in other research, such as the creation of the first World Atlas of artificial NSB [20] in which light propagation models are used in conjunction with satellite radiance data and other similar ones [21]. Garstang’s model is a good approximation, but results can be improved [22] and more complex models have been developed in this area. In 2007, Miroslav Kocifaj [13] proposed a light-pollution model which considers cloudy and cloudless night skies. As this author noted, common models are focused on astronomical observations, which are usually carried out in cloudless conditions so that parameters such as altitude and spectral reflectance of a cloud layer are contemplated. The same author proposed further theoretical models for light pollution under clear sky conditions, which were successfully verified with experimental data [23]. It was concluded that except for big cities nearby or multiple bright cities around the measuring point, urban light could be deducted considering a single scattering. Later, a sky glow model was published for determining the city’s emission function, which was verified with synthetical data, achieving consistent results [24]. Some restrictions of this model are that only clear sky conditions can be considered and radiance measurements should be in a range of 1–2 city diameters and several tens of city diameters.

These generalist physically based models can be used in multiple scenarios, such as the preliminary evaluation of new observatories, however, the continuous change of local conditions does not permit them to establish an accurate short-term forecast. In fact, as J. Slingo & T. Palmer stated [25], minimal changes in weather and climate predictions can dramatically affect the solution so that predictions must be based on probabilistic models instead of a single deterministic solution.

### 2.2. Probabilistic Model

**Figure 1:**
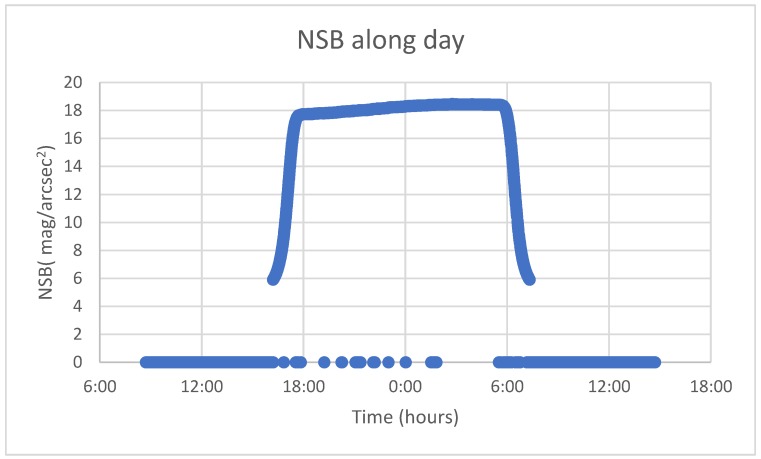
NSB curve of a random day in 2017 taken from the station at the National Astronomical Observatory of Japan. During diurnal hours, NSB values cannot be measured, while during nocturnal hours, NSB values rise, becoming stabilised and decreasing again.

To the best of our knowledge, there is no literature available on the use of probabilistic models applied to NSB time series forecasting. For this reason, solar radiation time series forecasting has been reviewed as a homologous case; as long as both temporal series are weather-dependent, the seasonal component plays a crucial role and the curves that represent its behaviour present large similarities (Figure 1). 

Traditionally, research in solar irradiance time series models has been developed using Autoregressive (AR), Autoregressive Moving Average (ARMA), Autoregressive Integrated Moving Average 9 (ARIMA) and Markov chain methods, which are based on probability estimation. However, these methods do not estimate well when stochastic components become higher, so other artificial intelligence (AI)-based methods have been applied in those cases, achieving better results [26].

Some authors proposed and tested several solar radiation models, such as ARIMA, Unobserved Components Models (UCM), regression models, transfer functions, neural networks and hybrid models, and have found that the ARIMA model shows better results than the rest because of its improved capacity to capture the diurnal cycle, though other methods show better results when using higher resolutions (around 5 min) [27]. Other investigations used historical data of solar radiation, temperature and sky conditions by applying fuzzy logic first and then neural networks in different sky conditions [26]. They achieved good results but concluded that the sky condition does not seem to have a significant impact on the forecast. Smart persistence, Artificial Neural Network (ANN) and random forest have been compared in order to predict solar radiation hourly within the subsequent six hours with the random forest showing the best performance [28]. As might be expected, the more hours one tries to forecast, the more inaccurate the prediction.

Some authors proposed a forecast method using solar data collected in three different locations with diverse meteorological variabilities [29]. They established a comparison of several methods, ARMA, ANN or regression tree-based methods, such as random forest, among others. It was concluded that Autoregressive Moving Average and multilayer perceptron were the most efficient for weak variabilities, for medium variabilities the Autoregressive Moving Average and multilayer perceptron were the most efficient, while for high variabilities, tree-based methods showed the best results.

Other research presented a short-term cloud forecast based on irradiance historical data collected in a radiometric network in Almeria (Spain) [30]. When applying a recurrent neural network known as Long Short-Term Memory (LSTM), they concluded that this method shows improvement in comparison with other common methods used for time series analysis.

## 3. Sky Quality Metre: Sensor Technology

For this research, NSB behaviour over the period of a year was modelled using field data. The characteristics of the technology sensor are presented in this section.

Research related to the use of artificial light during night periods and its consequences has become far more common in recent years. This trend “can be attributed to several factors, including: recognition of the impacts of artificial light on ecology and health, increasing amounts of artificial light in the environment, improved quality of imagery from space, the current global change in lighting technology, and increasing quality, ease, instruments, and methods for measuring light at starlight intensities” [31]. The most common indicator of the night darkness condition is given by the NSB, which refers to the narrow-band spectral radiation of the night sky generally measured in the astronomical magnitude system, instead of SI standard units, mag/arcsec^2^ (magnitude per square arc-second) [21]. This is a logarithmic scale in which values increase with darker skies. 

The SQM (Sky Quality Metre) is a commercially available NSB photometer with a pocket-size design that allows the quantification of the quality of the night sky [32]. This is a portable low-cost device originally developed by the Canadian manufacturer Unihedron with the aim of bringing to the general public, mostly amateur astronomers, the possibility of quantifying the sky conditions [31,32], however, it has been widely used for astronomy-related research [31,33,34,35]. The SQM “determines the amount of light scattered in the direction of the observer by the air column located above” [33], thus atmospheric conditions affect the measurement as well as the impacts from natural sources such as moonlight and other celestial bodies. It is known for having a rough linear response between 6 and 19 mag/arcsec^2^, its deviation is under 2.6%, the field of vision is about 20 degrees and the maximum measurable value is 22 mag/arcsec^2^, but it is not especially designed to measure properly in extremely low intensity environments [36]. As a reference, the darkest place on earth shows values of about 22 mag/arcsec^2^, values beyond 19 mag/arcsec^2^ may be found in rural areas, while bright cities show values between 16 and 17 mag/arcsec^2^ (more examples are collected in the cited references) [31,33,34,35,36].

The equipment is composed of a crystalline silicon (c-SI) photodiode TSL237 manufactured by Texas Advance Optoelectronic Solutions (TAOS), which combines a silicon photodiode and a current-to-frequency converter. The output signal is a square signal whose frequency is proportional to the intensity of the incident light. Additionally, it is equipped with an infrared blocking filter (HOYA CM-500) and only visible light is measurable [34,37]. Among multiple versions, the SQM-LE model is provided with an Ethernet connection, which allows measuring and saving data to a terminal. Regarding the measuring stability of the SQM, the device has been used for long periods of time, resulting in a small annual drift [38]. Moreover, periodical maintenance tasks are usually focused on the glass window cover, more than on the optical sensor [39]. 

One limitation of the SQM is that it gives an average value of the receiving light in the measurable frequency range, no matter what the colour of the light is. Sánchez de Miguel et al. identified that SQM readings vary depending on the colour of the receiving light, and although these authors conclude that SQM is a good instrument, they suggest that colour of light should be considered in the light pollution studies [40]. In addition, the increasing use of solid-state lighting technology (LED) for street illumination has led to an increase of “blue” light pollution against the “orange” light generated from classical high-pressure sodium lamps, which may cause a variation in the perception of NSB measured by the SQM [41,42].

Other authors have proposed some improvements to the original design, which is the case of TESS-W (Telescope Encoder Sky Sensor-Wireless). This device was created within the EU-funded STARS4ALL project [43] and proposes the addition of a dichroic filter, which is a very accurate colour filter, with the aim of extending the band pass to the red range [44]. Additionally, a WIFI module was included so that readings may be taken from a moving vehicle. Another case is the PePSS (Photomultiplier-equipped Portable Sky Scanner) developed by the Slovak Academy of Sciences and Comenius University, which uses a sophisticated photomultiplier technology that is able to read in extreme dark conditions [36].

Other one-dimension alternatives, such as dark sky metre phone applications which use the camera device or solar-cell-based light metres based on the measurements of photoelectric currents, may also be used for general NSB measurements [31]. Similarly, there exists two-dimensional equipment based on image capturing. Depending on the application, different kinds of camera are available; as an example, commercial digital cameras have been used for tracing the dynamics of skyglow by differential photometry, with the conclusion that it is a remarkable tool for this application [45]. In this regard, further analysis and comparison were carried out in a later paper with several light pollution measurement techniques (satellite image, one-dimension devices, image-based measurement), concluding that vertical plane images provide valuable additional information in comparison with other all-sky imagery [46]. 

**Figure 2:**
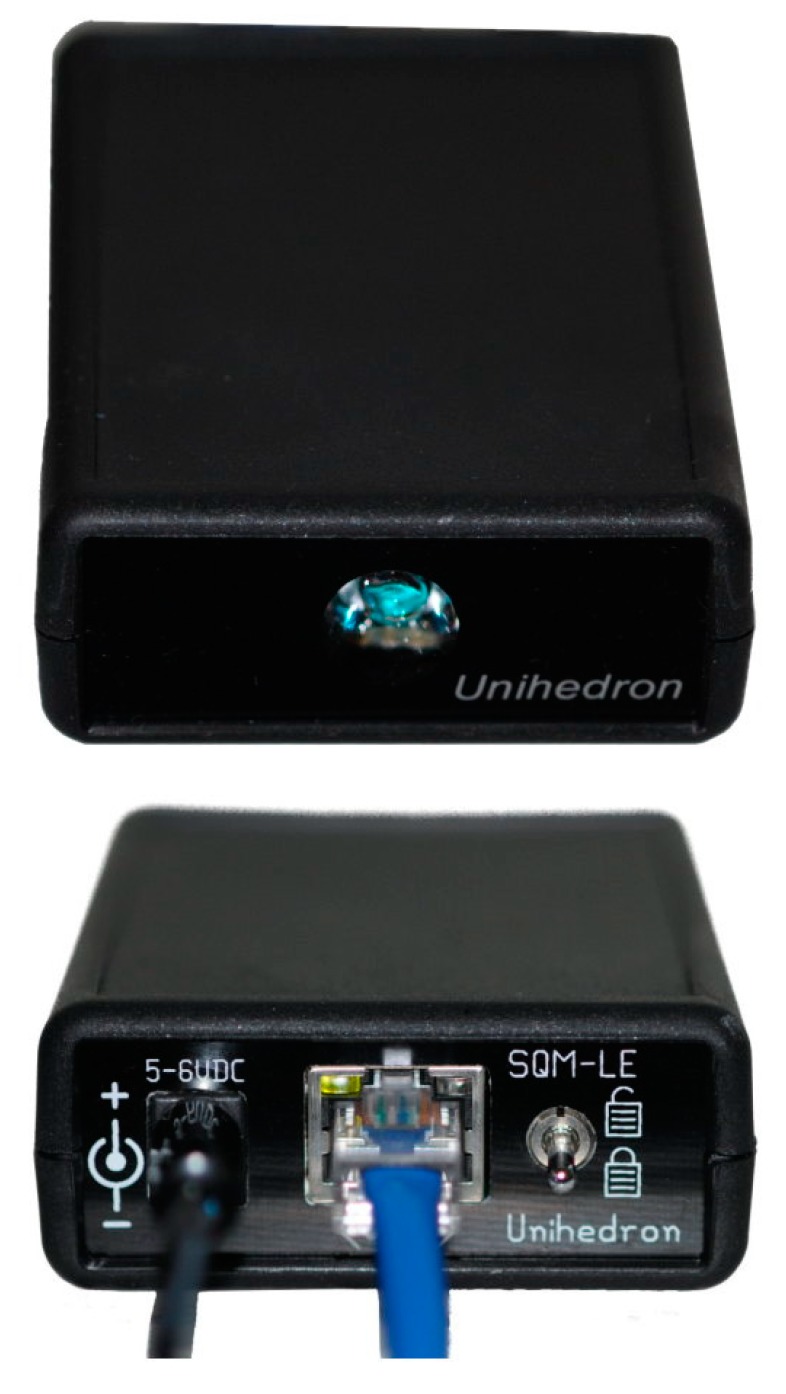
Sky Quality Metre-LE, source: Unihedron company.

In the context of this research, SQM equipment is more suitable for tourism destinations in terms of cost and ease of installation and its accuracy is precise enough for forecasting the night sky quality, more so in suitable destinations for astrotourism, which are usually in remote areas. The NSB dataset measured with an SQM-LE (by Unihedron) network (Figure 2) provided by the Globe at Night programme was used for training and testing a probabilistic model.

## 4. Methodology

The National Optical Astronomy Observatory (NOAO) of the United States of America and the Globe Project developed the Globe at Night programme in March 2016 with the aim of encouraging “citizen-scientists around the world to contribute simple unaided-eye observations on sky-brightness to a growing global database” [47]. According to this statement, The National Science Foundation (NSF) funded the purchase of 135 sky quality metres, which were distributed along several states in the USA, as well as another five countries, so that citizen-scientists could be trained to take the readings and record them online. 

More recently, the *Globe at Night-Sky Brightness Monitoring Network* (GaN-MN) project was created, which is an extension of the original Globe at Night project, with the aim of establishing a global sensor network for long-term monitoring of light pollution around the world using an SQM. Sky brightness is measured every 30 s and recorded in an open dataset available at the Globe at Night website. Every month, data from the previous six months are released.

For this research, data released on the Globe at Night website was used for training and testing a probabilistic model. In this section, authors present the pretreatment of such data, as well as modelling techniques used for NSB forecasting.

### 4.1. Understanding Data and Pretreatment

The open dataset released by the Globe at Night contains several variables such as sensor ID, NSB, temperature, date and others from multiple stations. This research aims to find an accurate method for NSB forecasting based on the study of the NSB univariate time series, thus only the date, local time and NSB measurements of a single station were used. Due to the fact that new stations are included every year, some datasets are provided with more historical data than others, so that a preliminary review was required. As a first approach, this methodology was applied to the station nicknamed “NAOJ”, which corresponds to the National Astronomical Observatory of Japan, located in Tokyo (coordinates: 35°40′31.4″N 139°32′15.7″E), because it was one of the most complete datasets; the whole year is included except for May, and data from 2016 and 2017 are available. As mentioned before, data were collected with a very high resolution (30 s) so it was necessary to average measurements into larger time slots. It must be considered that SQM measures sky brightness at a single point; hence it is exposed to rapid variation due to instantaneous external factors, which add noise to data and have a negative impact on the model. Additionally, in this kind of time series, it is recommended to limit the dataset according to some specific hours in which the variables to be predicted take a significant value. As an example, for solar radiation, it is common to restrict data to diurnal hours [26,30]. In this case, only nocturnal hours were considered, from 23 p.m. to 7 a.m. the next day.

**Figure 3:**
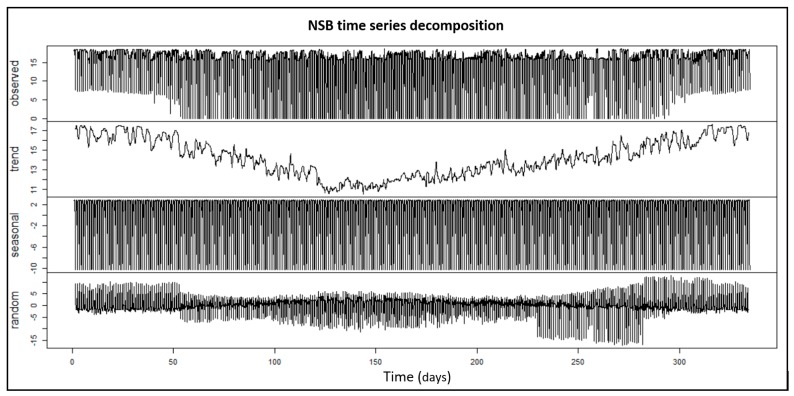
Additive decomposition for NSB time series for the whole of 2017 taken in the National Astronomical Observatory of Japan.

After these considerations, the result was an NSB univariate time series for the whole year (excluding May), which could be decomposed into three single time series. Decomposition aims to segregate the original time series into distinct components with different characteristics in order to analyse each one individually, therefore, it is a helpful analytical tool which allows finding some relationships that are not obvious in the original time series (Figure 3). An additive decomposition can be represented as the sum of three independent time series:

(1) $$y=\;y_{t}+y_{s}+y_{r}$$

in which:

$y_{t}$: Trend–cycle component
$y_{s}$: Seasonal component
$y_{r}:$ Random variation

The trend component indicates that NSB values decrease during the summer while registering the highest values in winter periods. This can be explained by two main reasons: NSB peak values are higher in winter than the rest of the year and the number of sun hours also decreases in this period. The seasonal component represents the time series with a fixed and known frequency, while the random variation component is the remaining value from the original series after extracting both previous components. It is noted that the random component has mainly positive values in winter and autumn periods and negative values in summer and spring periods, which is because the solar hours are not the same throughout the year and because values are only considered from 23 p.m. to 7 a.m. the next day.

### 4.2. Model Development

**Figure 4:**
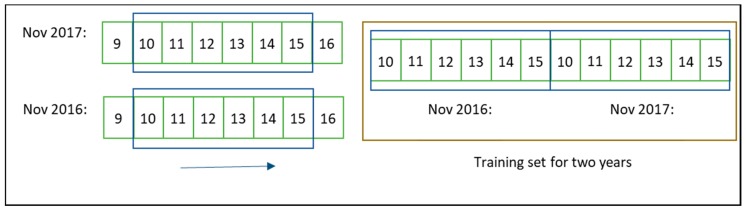
Example of sliding window with data from 2016 and 2017.

Before applying any method, data were preprocessed using the sliding window technique; this means that the training phase was carried out with data within a specific number of days prior to those intended for the forecast. Therefore, data was segmented according to a specific number of days before a date. This window is moved along the year so that new values can be forecast. Instead of taking a whole dataset, this technique allows using data near the day intended for the forecast, so that the data are more consistent, as long as conditions are similar. As an example, if the intention were to forecast the 16th of November, data from 10th of November (five days before) would be used in the training phase (Figure 4). Full data sets were available for 2016 and 2017 (except for May) and consequently, the sliding window technique can be set with data from a single year that is to be forecast or by adding the same window period for previous years. With the aim of establishing a proper comparison, both options were tested using several sliding windows. 

From the multiple models reviewed in the previous section, for this first approach, Autoregressive Integrated Moving Average (ARIMA) and Artificial Neural Network (ANN) were selected. As stated above, the ARIMA model has been widely used in the fields of nature and economics, as well as other time-varying series, and its forecast relies on past values as well as previous error terms [48]. This model is a combination of autoregressive models (AR) and moving average models (MA) with an additional parameter which represents the number of times that observations are differentiated [48,49]. In general terms, an ARIMA (p,d,q) model can be mathematically denoted as follows:

(2) $$\emptyset_{p}\left(B\right){\left(1-B\right)}^{d}z_{t}=\;\theta_{q}\left(B\right)a_{t}$$

where:

$\emptyset_{p}$ are unknown coefficients associated with AR model
$\theta_{q}$ are unknown coefficients associated with MA model
$z_{t}$ is the original time series
B is the backward shift operator ($Bz_{t}$ is the value of time series at  t−1)
d is the integrating order
$a_{t}$ is the white noise series

For each run, the configuration of the ARIMA model was established by multiple trial, looking for the one which presented the best Akaike Information Criterion (AIC). AIC is one of the most popular criteria for estimating the quality of statistical models given by:

(3) AIC=−2Log(Ɫ)+2m

where:

Log(Ɫ): is a measure of the model fit.
m: is the number of parameters estimated in the model

**Figure 5:**
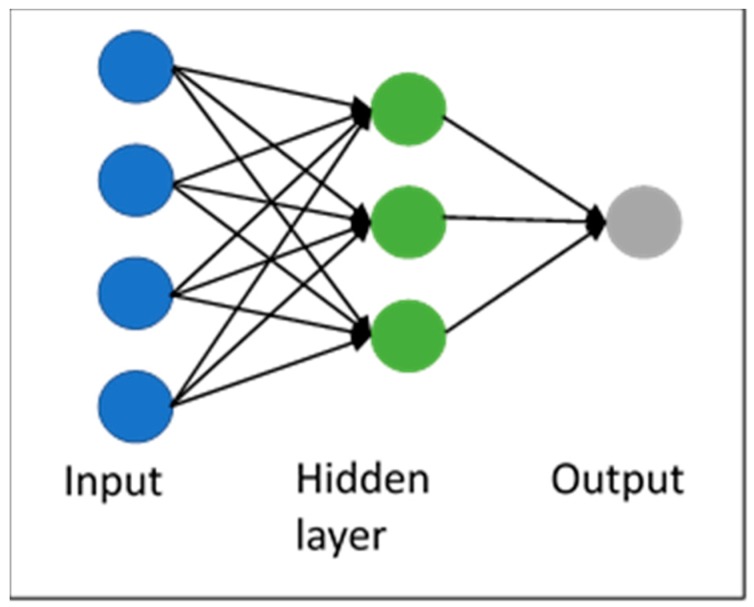
ANN conceptual diagram.

Additionally, feed-forward neural network was used for modelling NSB. This biologically inspired model has been widely used in time series forecasting [50] and it is composed of a specific number of nodes in each layer. Each layer receives as input the output from the previous layer in such a way that the input in each node is a weighted linear combination of the results of the previous layers (Figure 5) [51]. The input to a hidden node can be mathematically expressed by the following equation:

(4) $$z_{j}=\;b_{j}+\overset{n}{\underset{i=1}{\sum }}w_{i,j}y_{i}$$

where:

b: represent the bias
$w_{i,j}$: represents the weights
$y_{j}$: represents the input values for this layer

A neural network also needs to be configured, however, there is not a procedure for setting its parameters, therefore, model configuration is usually carried out by trial and error [52]. For the case of time series, pass values can be used as input values for modelling, which is known as Neural Network Autoregression model (NNAR) [51] and it is denoted as $NNAR{\left(p,\;P,\;k\right)}_{m}$; where p refers to the seasonal past values inputs; P refers to nonseasonal past values inputs and k refers to the number of hidden neurons in the hidden layer. The function NNETAR implemented in the forecast package for R statistical software [53] can build an NNAR model with only one single hidden layer [51]. The training phase is run 25 times using random stating values, so that the final model is the average of previous trains. Although it is a simple neural network configuration, some investigations note that this model performs better than other more complex ones [54].

## 5. Results

ARIMA and NNAR models were run using data from 2017 (Table 1) but also from 2017 and 2016 (Table 2). The subsequent 8 h (night hours) were forecast every 15 min and compared with real data. Several sliding windows were tested in order to find the one that showed better performance. 

**Figure 6:**
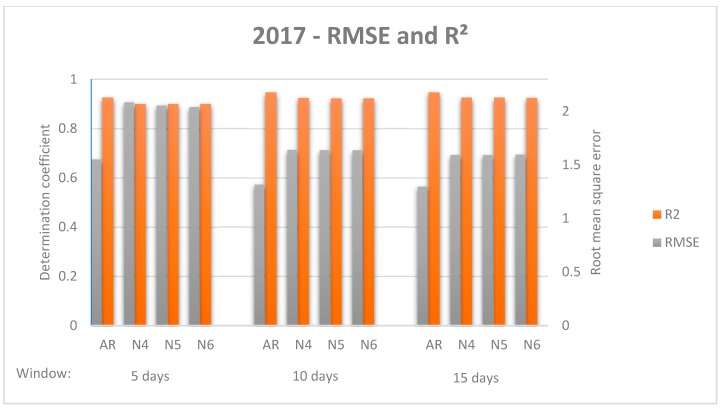
Comparison of forecasting models with different windows for data 2017. Root mean square error and coefficient of determination for each model and window are shown.

**Figure 7:**
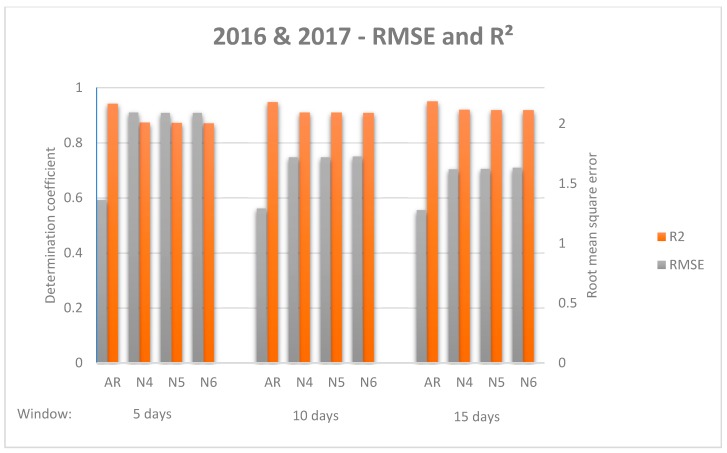
Comparison of forecasting models attending different windows for data 2016 & 2017. Root mean square error and coefficient of determination for each model and window are shown.

While there was no standardised evaluation measurement for comparing model performance [28], the decision was taken to use the Root Mean Square Error (RMSE), as it is one of the most commonly used measures in forecasting literature [54]. However, some authors indicate that Mean Absolute Error (MAE) “is a more natural measure of average error” and recommend its use [55]. For establishing a proper comparison, both indicators were used, as well as standard deviation and the coefficient of determination. Additionally, RMSE and R^2^ are represented (Figure 6 and Figure 7) to facilitate comparative analysis.

As a general impression, all models improve their results when the sliding window becomes longer; while it is true that a significant difference is found from the first window to the second, there is no such difference from the second window to the third. Similarly, NNETAR models improve results when the number of neurons increases, but there is no significant difference among them. In all cases, ARIMA models outperform NNETAR models, however, the ARIMA models respond better when data from both 2016 and 2017 are used, whereas the best results of the NNETAR model are found when just 2017 data are used. Considering both data from 2017 and 2016 & 2017, the dispersion measure increases for NNETAR models when the sliding window is longer. For the ARIMA model, standard deviation increases with the increase of the sliding window for 2016 & 2017 data, whereas it decreases for 2017 data.

## 6. Conclusions and Future Research

In this research, two probabilistic methods have been proposed for modelling night sky brightness (NSB), achieving promising results. Results show that in all cases, the ARIMA model outperformed the Artificial Neural Network model, because the ANN configuration was used with only one single layer, therefore, a more complex configuration could be used in further research. However, despite the ARIMA model providing better goodness of fit, ANN showed low dispersion in terms of standard deviation. In the same way, ARIMA showed better performance using data from 2016 and 2017, while ANN improved its results using data only from 2017. This suggests that ARIMA can better assume the variances in the time series, but it affects the variability of the outputs.

This NSB short-term forecast is expected to provide visitors with sky quality information so that they can plan their celestial tour. It should be considered that places usually suitable for celestial tourism tend to be located in remote areas and visitors must travel long distances expecting to be lucky. If tourists were better informed with an accurate NSB forecast, they would be able to plan their trip accordingly and not waste their holiday time. In this regard, customer satisfaction is crucial for encouraging client loyalty, but at the same time, customers may consider NSB forecasts as an additional value as long as they are not a common facility. 

Future research may contemplate the use of other local parameters (e.g., ambient temperature/humidity or sky conditions) as predictors or even ARIMA–ANN hybrid models, such as Díaz-Robles et al. [56]. This research has focused on forecasting the subsequent 8 h, but longer forecast periods may be tested. In the same manner, in this first approach, a resolution of 15 min was adopted, however, other proposals may modify this parameter.

In this kind of time series, periods in which values are known or do not contribute to the model are usually removed (night periods in the case of solar radiation or diurnal hours in the case of NSB). This paper considered data from 23 p.m. to 7 a.m., but this period could be adapted according to the season.

On the other hand, we have used additive decomposition for a better understanding and analysis of the NSB time series behaviour, but once decomposed, trend cycles and seasonal components could be modelled separately from the random component, in which more complex methods, such as multilayer ANN, could be used.

The methodology presented in this paper has achieved very good results of NSB estimations, however, further work should include other stations in different locations for which parameters such as resolution or night period must be considered or architectural parameters for ANN must be specifically studied.

To the best of our knowledge, this is the first time that a probabilistic model has been applied to NSB. This first model approach opens new promotional possibilities in the astrotourism sector, as well as encouraging further study into the modelling and data treatment of NSB.

## Figures and Tables

**Table 1:** Forecasting results using data from 2017: Standard deviation, determination coefficient, root mean square error and mean absolute error for ARIMA and ANN.

Data 2017					
Window	Model	Sd	R^2^	RMSE	MAE
**5 days**	ARIMA	5.697	0.927	1.553	1.004
	NNAR (K = 4)	4.289	0.901	2.088	1.548
	NNAR (K = 5)	4.345	0.901	2.058	1.526
	NNAR (K = 6)	4.381	0.901	2.041	1.513
**10 days**	ARIMA	5.658	0.947	1.317	0.871
	NNAR (K = 4)	4.970	0.925	1.641	1.190
	NNAR (K = 5)	5.004	0.924	1.638	1.187
	NNAR (K = 6)	5.025	0.923	1.637	1.185
**15 days**	ARIMA	5.663	0.948	1.300	0.851
	NNAR (K = 4)	5.072	0.927	1.594	1.149
	NNAR (K = 5)	5.101	0.926	1.595	1.149
	NNAR (K = 6)	5.119	0.925	1.597	1.149

**Table 2:** Forecasting results using data from 2016 and 2017: Standard deviation, determination coefficient, root mean square error and mean absolute error for ARIMA and ANN.

Data 2016 & 2017					
Window	Model	Sd	R^2^	RMSE	MAE
**5 days**	ARIMA	5.629	0.943	1.363	0.889
	NNAR (K = 4)	4.723	0.874	2.095	1.574
	NNAR (K = 5)	4.757	0.873	2.090	1.570
	NNAR (K = 6)	4.774	0.872	2.092	1.571
**10 days**	ARIMA	5.670	0.949	1.293	0.847
	NNAR (K = 4)	5.182	0.911	1.720	1.279
	NNAR (K = 5)	5.205	0.910	1.722	1.277
	NNAR (K = 6)	5.217	0.909	1.728	1.280
**15 days**	ARIMA	5.693	0.951	1.278	0.837
	NNAR (K = 4)	5.354	0.921	1.620	1.194
	NNAR (K = 5)	5.371	0.920	1.625	1.196
	NNAR (K = 6)	5.380	0.919	1.632	1.199
